# *Plasmodium falciparum* artemisinin resistance monitoring in Sabah, Malaysia: in vivo therapeutic efficacy and *kelch13* molecular marker surveillance

**DOI:** 10.1186/s12936-018-2593-x

**Published:** 2018-12-10

**Authors:** Matthew J. Grigg, Timothy William, Kim A. Piera, Giri S. Rajahram, Jenarun Jelip, Ammar Aziz, Jayaram Menon, Jutta Marfurt, Ric N. Price, Sarah Auburn, Bridget E. Barber, Tsin W. Yeo, Nicholas M. Anstey

**Affiliations:** 1 Global and Tropical Health Division, Menzies School of Health Research and Charles Darwin University, Casuarina, PO Box 41096, Darwin, NT 0811 Australia; 2 Infectious Diseases Society Sabah-Menzies School of Health Research Clinical Research Unit, Kota Kinabalu, Sabah Malaysia; 3 Jesselton Medical Centre, Kota Kinabalu, Sabah Malaysia; 4 Clinical Research Centre, Queen Elizabeth Hospital, Ministry of Health, Kota Kinabalu, Sabah Malaysia; 5 QIMR Berghofer Medical Research Institute, Brisbane, QLD Australia; 6 Sabah Department of Health, Ministry of Health, Kota Kinabalu, Sabah Malaysia; 7 Vector Disease Sector, Disease Control Division, Ministry of Health, Kuala Lumpur, Malaysia; 8 Centre for Tropical Medicine and Global Health, Nuffield Department of Clinical Medicine, University of Oxford, Oxford, UK; 9 Lee Kong Chian School of Medicine, Nanyang Technological University, Singapore, Singapore; 10 Communicable Disease Centre, Institute of Infectious Diseases and Epidemiology, Tan Tock Seng Hospital, Singapore, Singapore

**Keywords:** *Plasmodium falciparum*, Malaria, *kelch*, K13, Artemisinin-resistance

## Abstract

**Background:**

Spreading *Plasmodium falciparum* artemisinin drug resistance threatens global malaria public health gains. Limited data exist to define the extent of *P. falciparum* artemisinin resistance southeast of the Greater Mekong region in Malaysia.

**Methods:**

A clinical efficacy study of oral artesunate (total target dose 12 mg/kg) daily for 3 days was conducted in patients with uncomplicated falciparum malaria and a parasite count < 100,000/µL admitted to 3 adjacent district hospitals in Sabah, East Malaysia. On day 3 and 4 all patients were administered split dose mefloquine (total dose 25 mg/kg) and followed for 28 days. Twenty-one *kelch13* polymorphisms associated with *P. falciparum* artemisinin resistance were also evaluated in *P. falciparum* isolates collected from patients presenting to health facilities predominantly within the tertiary referral area of western Sabah between 2012 and 2016.

**Results:**

In total, 49 patients were enrolled and treated with oral artesunate. 90% (44/49) of patients had cleared their parasitaemia by 48 h and 100% (49/49) within 72 h. The geometric mean parasite count at presentation was 9463/µL (95% CI 6757–13,254), with a median time to 50% parasite clearance of 4.3 h (IQR 2.0–8.4). There were 3/45 (7%) patients with a parasite clearance slope half-life of ≥ 5 h. All 278 *P. falciparum* isolates evaluated were wild-type for *kelch13* markers.

**Conclusion:**

There is no suspected or confirmed evidence of endemic artemisinin-resistant *P. falciparum* in this pre-elimination setting in Sabah, Malaysia. Current guidelines recommending first-line treatment with ACT remain appropriate for uncomplicated malaria in Sabah, Malaysia. Ongoing surveillance is needed southeast of the Greater Mekong sub-region.

**Electronic supplementary material:**

The online version of this article (10.1186/s12936-018-2593-x) contains supplementary material, which is available to authorized users.

## Background

The spread of *Plasmodium falciparum* artemisinin resistance from Southeast Asia threatens global malaria elimination efforts [1, 2]. To date public health concerns have focused on the significant danger of artemisinin-resistant *P. falciparum* spreading westwards from Greater Mekong region into the Indian subcontinent and onwards to sub-Saharan Africa, where the majority of the global burden of disease exists [3, 4]. However, monitoring for the south-eastward spread of artemisinin resistance to other endemic countries in Southeast Asia, many of which are approaching elimination of *P. falciparum* and other human-only *Plasmodium* species, is also essential to ensure that the significant public health gains in this region are not lost [3].

The discovery of molecular markers associated with *P. falciparum* artemisinin resistance on the *kelch* gene on chromosome 13 (*kelch13*) [5] has provided an important tool for effective molecular surveillance [1, 2, 6], which can help define the geographical spread of resistant parasites, and guide targeted malaria public health activities. Early detection of *P. falciparum* artemisinin resistance also facilitates optimal treatment strategies to protect the concurrent development of resistance in longer-acting artemisinin-based combination therapy (ACT) partner drugs, such as piperaquine [7–9].

In Malaysia, the reported number of falciparum malaria cases has decreased from around 5000 in 2010 to less than 20 in 2017; with progress on track to meet the national WHO elimination goal for human-only malaria by 2020 [3]. Malaysia borders Thailand where artemisinin-resistant *P. falciparum* is established. At the time of study design, no previous studies had evaluated the presence of artemisinin resistance in Malaysia, particularly in East Malaysia on the island of Borneo where the majority of *P. falciparum* transmission occurs [10, 11]. To determine whether artemisinin-resistant *P. falciparum* was present in Sabah, Eastern Malaysia, a clinical efficacy study of oral artesunate was conducted with complementary parasite molecular studies to genotype *kelch13* polymorphisms associated with artemisinin resistance.

## Methods

### Clinical efficacy study

Patients were enrolled from October 2012 to February 2016 at 3 adjacent district hospital sites at Kudat, Kota Marudu and Pitas in northwest Sabah, Malaysia. Patients weighing more than 10 kg and up to 65 years of age were enrolled if they had acute uncomplicated malaria, confirmed by positive blood smear for asexual forms of *P. falciparum* on hospital microscopy, with a parasite count between 1000 and 100,000/µL, a positive *P. falciparum* histidine-rich protein 2 (Pf-HRP2) result on malaria rapid diagnostic test (CareStart™ Pf/Pan, Access Bio, USA), a documented temperature > 37.5 °C or a history of fever in the last 48 h. Written informed consent was obtained in all patients, including from a relevant parent/guardian for those < 18 years of age. Patients were excluded if there were any WHO-defined clinical or laboratory signs of severe malaria [12], or they had a haemoglobin concentration at presentation < 7 g/dL, had received artemisinin-containing therapy in the previous 7 days, had a concurrent acute illness was present, had a previous splenectomy, or, for females, if they were pregnant or breastfeeding. Subsequent diagnostic PCR was conducted for all microscopy positive patients [13, 14]; only those with *P. falciparum* confirmed infections were included in the final analysis.

Patients received oral artesunate alone daily for 3 days [target dose 4 mg/kg, using 75 mg/5 kg dose-weight banding increments (Table 1)] [1], followed by oral mefloquine (target dose 25 mg/kg, split in 2 doses of 15 mg/kg and 10 mg/kg) over 2 subsequent days (Artequin^®^, Novartis, Switzerland). Demographic, clinical and laboratory data were collected at presentation and then daily during hospital admission. Blood slides for microscopy were obtained 6-hourly until a minimum of 2 consecutive negative results were recorded. Whole blood samples were collected at enrolment, then at 24, 48, and 72 h after treatment, and at final follow-up 28 days after enrolment.

**Table 1:** Oral artesunate dose-bodyweight banding

Dose band	Bodyweight (kg)	Total dose (mg)	Number of patients	Dose (mg)/bodyweight (kg) (mean, SD)
1	10–16	150	4	11.1 (2.6)
2	17–22	225	5	11.6 (1.0)
3	23–28	300	3	11.8 (1.1)
4	29–34	375	5	11.9 (0.8)
5	35–41	450	5	12.0 (0.6)
6	42–47	525	4	11.5 (0.3)
7	48–53	600	11	11.9 (0.5)
8	54–59	675	6	12.2 (0.3)
9	60–65	750	4	12.0 (0.4)
10	66–72	825	1	11.4 (–)
11	73–77	900	0	–
12	78–85	975	1	11.9 (–)

### *kelch13* molecular surveillance

Molecular markers previously associated with *P. falciparum* artemisinin resistance were evaluated using PCR to detect 21 single nucleotide polymorphisms on the *kelch13* gene (see Additional file 1), as previously described [5, 15]. Additional patients evaluated for *kelch13* mutations included those with falciparum malaria excluded from the clinical trial, but who were enrolled in a concurrent clinical malaria study at the same sites and during the same study period [16], as well as patients with falciparum malaria enrolled in an ongoing prospective malaria study at the state tertiary referral hospital in Kota Kinabalu from 2010 to 2016 [17].

Data were entered into Epidata (version 3.1) and analysed using Stata (version 12). Microscopic asexual parasitaemia and gametocytaemia were calculated from thick blood smears [18]. Best-fit linear or tobit regression models were used to estimate the curve of log_e_ parasite counts over time [19].

## Results

### Clinical efficacy study

**Fig. 1:**
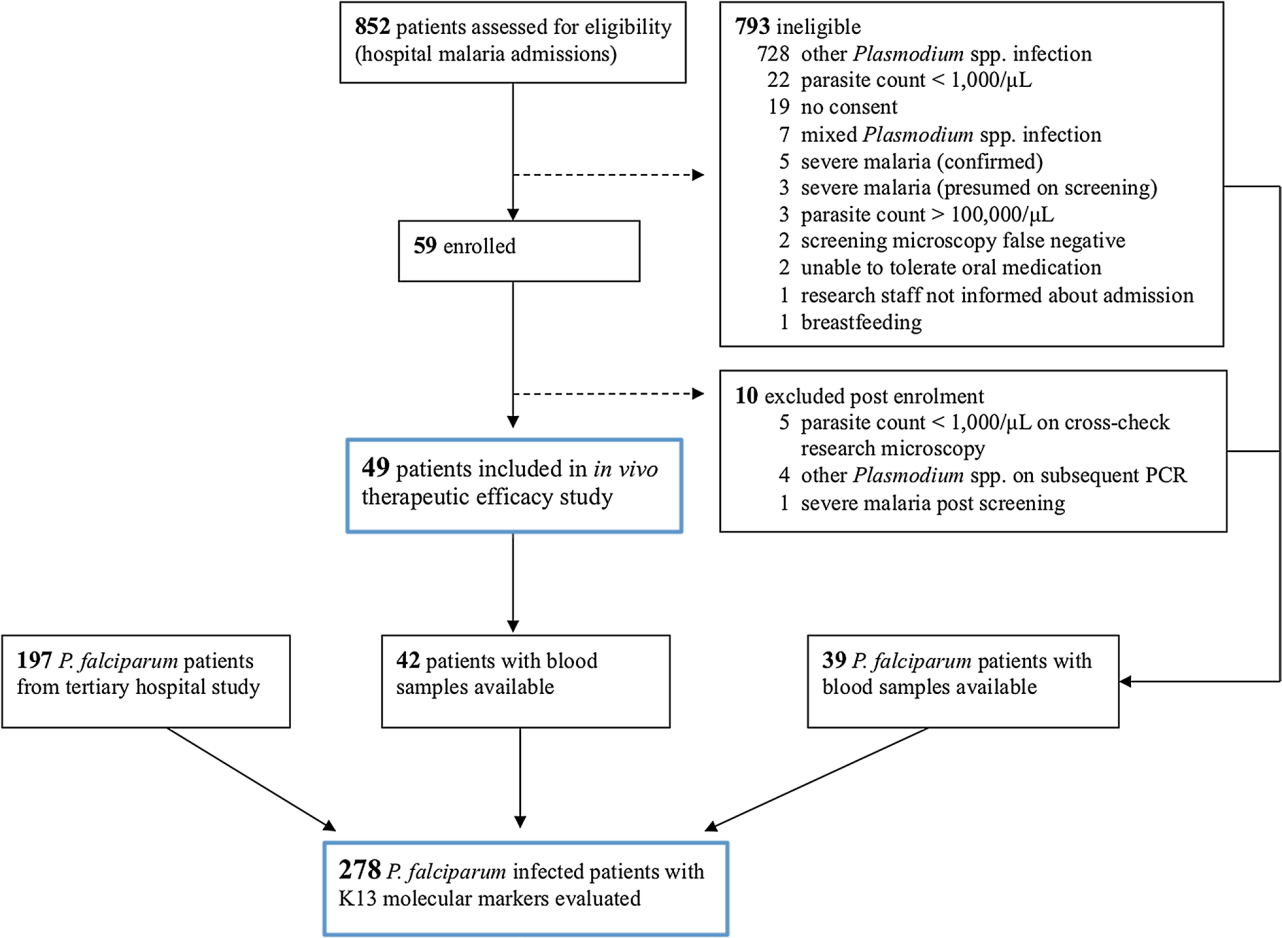
Patient enrolment flowchart

852 patients with malaria were admitted to the study site hospitals, of whom 59 patients with *P. falciparum* on screening microscopy were enrolled into the study (Fig. 1). Of these patients, 49 with *P. falciparum* infection met the study inclusion and exclusion criteria and were included in the final analysis (Table 2). The median age was 18 (IQR 11–31), with 14 (29%) children aged 12 years or less. There were 7 (14%) patients with a previous self-reported history of malaria requiring hospital admission, and the median duration of preceding fever was 5 days. The most common presenting symptom was headache (92%), followed by chills or rigors (67%), vomiting (49%), and muscle and joint pain (45%). At presentation, the geometric mean parasite count was 9463/µL (95% CI 6757–13,254), and 24 (49%) patients had anaemia (using WHO age-related criteria [20]), 3 (6%) patients had acute kidney injury (KDIGO criteria [21]) and a single patient was G6PD deficient. The mean oral artesunate dose per bodyweight was 11.8 mg/kg (SD ± 0.9), with no statistically significant difference demonstrated in the final total dose per bodyweight received between those in different dose-weight bands (Table 1).

**Table 2:** Demographic and clinical data

Patient characteristic	*P. falciparum* (n = 49)
Age, years	
Median (IQR)	18 (11–31)
Range	3–62
Children (age ≤ 12 years), n (% total)	14 (29)
Male gender, n (%)	38 (78)
Previous malaria (self-reported), n (%)	7 (14)
History of chronic disease, n (%)	0 (0)
Days of fever, median (IQR)	5 (3–6)
Symptoms on enrolment, n (%)	
Rigors/chills	33 (67)
Headache	45 (92)
Vomiting	24 (49)
Abdominal pain	15 (31)
Diarrhoea	6 (12)
Cough	16 (33)
Shortness of breath	5 (10)
Myalgia	21 (43)
Arthralgia	22 (45)
Examination findings on enrolment	
Temperature, °C, median (IQR)	37.3 (37.0–38.2)
Fever, temp ≥ 37.5 °C, n (%)	20 (41)
Systolic blood pressure, mmHg, median (IQR)	111 (99–120)
Heart rate, beats/min, median (IQR)	96 (83–106)
Respiratory rate, breaths/min, median (IQR)	22 (20–24)
Oxygen saturation, %, median (IQR)	99 (98–100)
Palpable liver, n (%)	11 (22)
Palpable spleen, n (%)	5 (10)
Rash, n (%)	
Laboratory findings on enrolment	
Parasite count, parasites/μL, median (IQR)	9924 (3466–24,276)
Parasite count, parasites/μL, range	1459–56,789
Parasite count, parasites/μL, geometric mean (95% CI)	9463 (6757–13,254)
Ring proportion, mean % (SD)	89 (0.24)
Haemoglobin, g/dL, median (IQR)	12.3 (11.0–13.9)
Anemia (baseline), n (%)*	24 (49)
G6PD deficiency present, n/N (%)	1/38 (97)
White blood cell count, × 10^3^/μL, median (IQR)	6.9 (5.6–8.9)
Neutrophil count, × 10^3^/μL, median (IQR)	4.1 (2.5–6.0)
Platelet count, × 10^3^/μL, median (IQR)	122 (57–190)
Thrombocytopenia (platelets < 150 × 10^3^/μL), n (%)	30 (61)
Creatinine, μmol/L, median (IQR)	69 (50–82)
Urea, mmol/L, median (IQR)	4.6 (3.3–5.8)
Bilirubin, μmol/L, median (IQR)	14.6 (9.3–24.0)
Glucose, mmol/L, median (IQR)	6.3 (5.5–7.1)
Albumin, g/dL, median (IQR)	34 (28–37)
ALT, IU/L, median (IQR)	30 (20–45)
Bicarbonate, mmol/L, median (IQR)	20.9 (20.0–22.8)
Acute kidney injury, n (%)	3 (6)
Blood cultures positive, n (%)^✝^	0/29 (0)

* Anemia based on WHO 2011 haemoglobin measurement criteria [20]: age 6–59 months (≤ 100g/dL), 5–11 years (< 115g/dL), 12–14 years (< 120g/dL), non-pregnant women ≥ 15 years (< 120g/dL), pregnant women (< 110g/dL), men ≥ 15 years (< 130g/dL)

^✝^Excluding results positive for skin contaminants

**Fig. 2:**
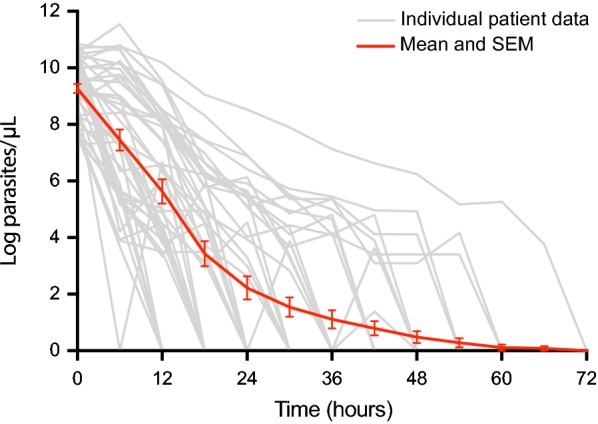
Parasite clearance

At 48 h after treatment 44/49 (90%; CI 95 78–97) of patients were negative for parasites microscopically on blood smear. All patients were negative by 72 h, and remained negative up to 28 days post-treatment (Table 3). The median parasite clearance time was 25.6 h (IQR 18.5–42), with the slowest clearance seen at the 72 h planned time-point in a patient with an initial parasite count at presentation of 44,622/µL (Fig. 2). In multivariable analysis including age, sex and artesunate dose per bodyweight, parasite count at presentation was the only independent predictor of parasite clearance time (*p *= 0.001).

**Table 3:** Parasitological response

Variable	Artesunate, n = 49
Parasitological response	
24 h	
Number negative	29
% (CI 95)	59.2 (44.2–73.0)
48 h	
n	44
% (CI 95)	89.8 (77.8–96.6)
72 h	
n	49
% (CI 95)	100 (92.7–100)
Parasite clearance time (PCT)	
Median hours	25.6
(IQR)[range]	(18.5–42)[7.9–73.9]
Slope of curve *(k)* for log_e_ Normalised parasite clearance*	
Mean *(k)* constant (CI 95)	0.26 (0.20–0.32)
Slope of curve *(k)* half-life	
Median hours (IQR)	2.7 (2.2–3.5)
Slope of curve (k) half-life > 5 h	
n (%)	3 (6.7)
Lag phase present	
n (%)	9 (20)
Lag phase duration	
Median hours (IQR)	7.7 (7.3–8.7)
PCT_50_	
Median hours (IQR)	4.3 (2.0–8.4)
PCT_90_	
Median hours (IQR)	10.8 (6.8–15.6)
PCT_95_	
Median hours (IQR)	13.4 (9.0–18.6)
PCT_99_	
Median hours (IQR)	19.7 (14.3–25.6)

* 45 (92%) had data at ≥ 3 time points allowing parasite clearance analysis

Detailed parasite clearance estimation (using WWARN methodology [19]) could be derived on 45 (92%) patients. Four patients were excluded as their parasite clearance was less than 12 h and insufficient for analysis. Of the 45 patients evaluated, the median time to 50% parasite clearance (PCT_50_) was 4.3 h (IQR 1.9–8.4), and time to 99% parasite clearance was 19.6 h (IQR 14.3–25.6). The mean slope of the log_e_ normalised parasite clearance curve was 0.27 (95% CI 0.24–0.30). The median half-life of the clearance slope was 2.7 h (IQR 2.2–3.5), with 3 (7%) of patients having a clearance slope half-life greater than 5 h. There was no difference in the median clearance slope half-life when comparing patients enrolled from each year during the study period.

### *kelch13* molecular surveillance

**Fig. 3:**
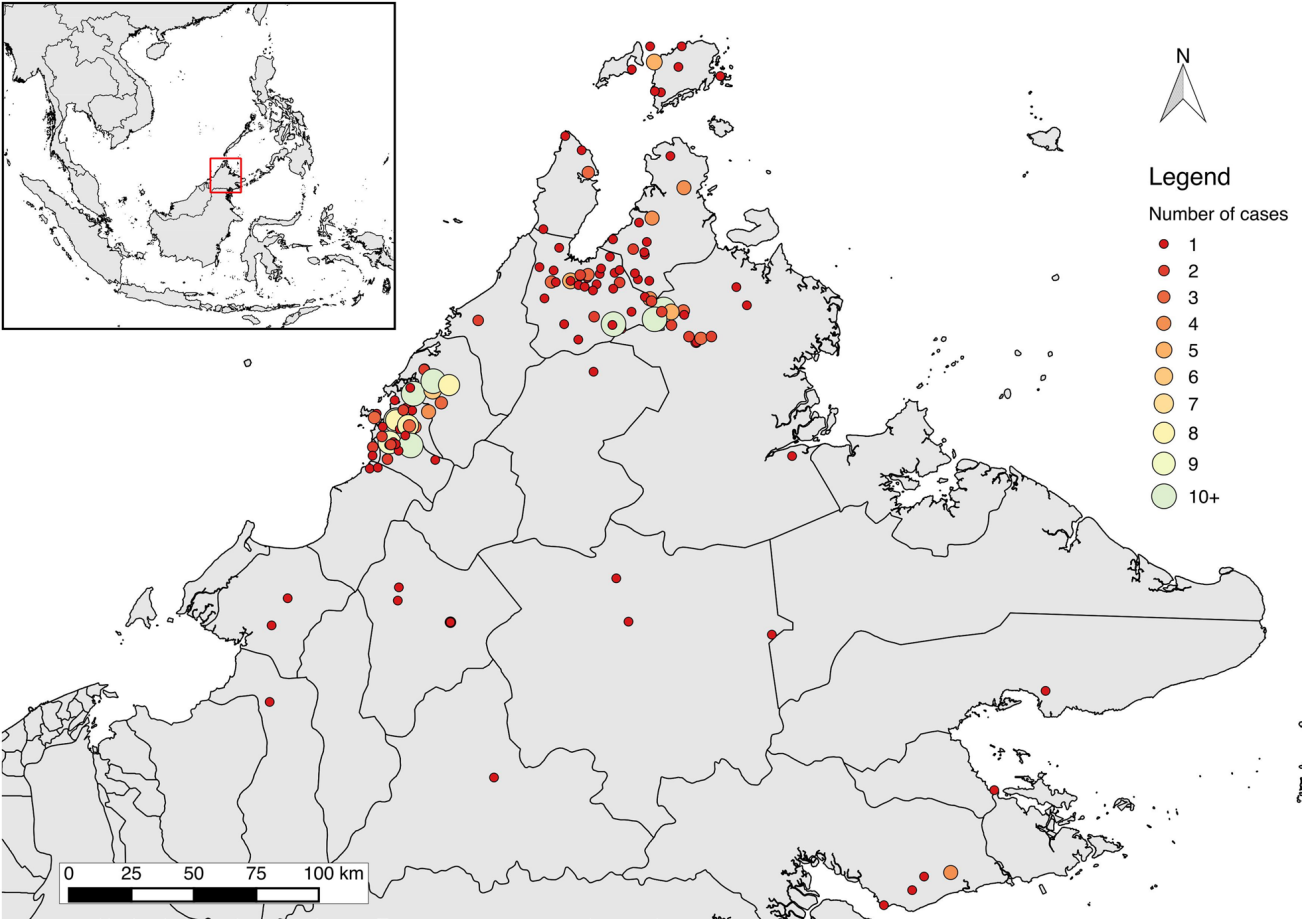
Geographical distribution of cases with K13 molecular markers evaluated

Molecular markers were conducted in 278 patients with falciparum malaria (Fig. 3), of whom 42 (86%) had also been enrolled in the clinical efficacy study. All *P. falciparum* isolates were wild-type for the 21 *kelch13* SNPs evaluated (GenBank Accession Numbers: MH721937–722214), including 98 (100%) of those subsequently tested for the F446I polymorphism (unpublished). Cases were predominantly distributed from the West Coast administrative division of Sabah (158 [58%]), followed by the northern Kudat division (111 [41%]), with 5 (2%) additional cases from the eastern Sandakan division (Fig. 3).

## Discussion

*Plasmodium falciparum* parasites causing malaria in Sabah, Malaysia remain sensitive to treatment with oral artesunate, with no evidence of delayed in vivo parasite clearance at 72 h [22], and an absence of known polymorphisms associated with artemisinin resistance in the *kelch13* propeller domain [1]. No WHO criteria for suspected or confirmed endemic artemisinin resistance were present [23]. Confirmation of ongoing efficacy to the major component of ACT supports the continuation of standard WHO recommended ACT regimens as the first-line treatment for uncomplicated falciparum malaria in Malaysia [24], as is currently indicated in national malaria treatment guidelines [25]. ACT remains highly efficacious and the first-line treatment for the other endemic *Plasmodium* species causing malaria in Malaysia [25], which are commonly misidentified using microscopic diagnosis [26]. This includes uncomplicated human malaria due to zoonotic transmission of the monkey parasite *Plasmodium knowlesi*, for which ACT has faster parasite clearance, lower risk of anaemia and earlier hospital discharge compared to chloroquine [27, 28], and also malaria caused by *Plasmodium vivax*, for which high-grade chloroquine resistance has been demonstrated for blood-stage infections in Sabah [29].

Results from this study are consistent with another study from Sabah, which reported only wild-type *kelch13* molecular markers using 17 SNPs from 50 *P. falciparum* isolates collected from 2008 to 2014 [30]. The absence of *P. falciparum* artemisinin drug resistance in Malaysia, either spread from imported cases from neighbouring countries harbouring resistant *P. falciparum* parasites, or de novo resistance arising independently, is likely due in large part to the effective Malaysian malaria public health programme [25]. Malaysian Ministry of Health malaria guidelines stipulate all patients with confirmed malaria by microscopy require admission to hospital for free treatment and management, in addition to distribution of long-lasting insecticide-treated bed nets and active case detection for household contacts of reported cases in endemic areas [3, 25]. ACT has been the first-line treatment for uncomplicated falciparum malaria since 2009, with centrally distributed artemether-lumefantrine (produced according to good manufacturing practice) preferentially used [25]. Widespread access to free supervised treatment has meant that counterfeit or poor quality artemisinin-based combinations, or partially completed courses of treatment, have not been a major concern, limiting the potential impact of these factors on the risk of independent resistance developing. Primaquine given as a single dose of 0.25 mg/kg for patients with normal G6PD activity for transmission blocking of gametocytes was also introduced in Malaysia in 2014, limiting the onward transmission risk and further drug exposure of parasites potentially developing artemisinin resistance.

Ongoing therapeutic efficacy monitoring for artemisinin derivatives should incorporate the use of *kelch13* molecular markers, as these allow earlier detection of resistance compared to parasitological measures [1]. At the time of study design, and prior to the discovery of the *kelch13* molecular markers, artemisinin resistance was best defined as the presence of delayed parasite clearance (microscopically positive for asexual *P. falciparum* at 72 h post-treatment with an ACT in those presenting with parasite counts < 100,000/µL) in ≥ 3% of a treated population [22]. However, this method has limitations as a regional strategy. Quantification of parasite clearance in countries using different artemisinin-based combination treatments are difficult to compare due to differences in the relative efficacy between the longer acting partner drugs. Initial efficacy of the ACT partner drug also masks the underlying development of early artemisinin resistance, leading to a higher risk of subsequent partner drug resistance [7, 8]. Conventional methods of measuring parasite clearance also exclude the initial highly variable lag phase in the first 6–12 h, which is impacted by the speed of action of the antimalarial and the life-cycle stage of parasites at the time of drug administration [31].

The ongoing decline in falciparum malaria cases in Sabah over the study period limited both the number of those able to be enrolled in the in vivo parasitological assessment, and also the overall number evaluated in the *kelch13* molecular study component. Falciparum malaria cases able to have *kelch13* marker surveillance conducted were predominantly from the western Sabah catchment area of the tertiary referral hospital in the state capital Kota Kinabalu. Although it is possible more remote interior areas were underrepresented, this area has both the highest population density and falciparum malaria transmission in Sabah, as well as significant numbers of official and unofficial migrants from surrounding falciparum-endemic countries.

Accurate evaluation of parasite clearance was possible by the study design utilising optimal dosing of oral artesunate (4 mg/kg) given in narrow weight bandings, with all patients receiving close to the target total dose of 12 mg/kg. In contrast, the first-line ACT used in Malaysia, artemether-lumefantrine, has an effective artemisinin derivative component dose of 1.8 mg/kg, given twice daily, with larger weight-bandings for standard WHO-recommended use known to result in lower efficacy among young children from Asia receiving ≤ 60 mg/kg total lumefantrine dose [32]. However, any potential differences in parasite clearance due to variation in dosing between the study design and standard ACT regimens in routine practice are not a result of underlying *P. falciparum* artemisinin resistance in this population given the absence of *kelch13* polymorphisms.

## Conclusion

*Plasmodium falciparum* artemisinin resistance was not evident in Sabah, Malaysia from in vivo parasite clearance, or surveillance of *kelch13* molecular markers. Current ACT regimens should continue to be used as the first-line blood-stage treatment for all *Plasmodium* species in Malaysia. With Malaysia approaching the elimination of falciparum malaria, ongoing molecular surveillance for artemisinin and partner drug resistance will be an important adjunctive tool in achieving and maintaining this goal. Wider surveillance for artemisinin and partner drug-resistant *P. falciparum* in other countries to the south and east of the Greater Mekong region will also be an important component in the success of their malaria elimination goals.

## Additional file

**Additional file 1.** *kelch13* molecular marker reference table.
