# Supplementary material for: Plasmodium falciparum artemisinin resistance monitoring in Sabah, Malaysia: in vivo therapeutic efficacy and kelch13 molecular marker surveillance
Source: Malar J. 2018 Dec 10;17:463. doi: 10.1186/s12936-018-2593-x (PMC6287347; doi:10.1186/s12936-018-2593-x)
Supplement: Supplementary file 1 — Additional file 1. kelch13 molecular marker reference table. [file 12936_2018_2593_MOESM1_ESM.docx]

**Additional file 1**: *kelch13* molecular marker reference table [15]

| Codon Position | Amino Acid reference sequence | Nucleotide reference sequence | Amino Acid mutant-type sequence | Nucleotide mutant-type sequence |
| --- | --- | --- | --- | --- |
| 446 | F | ttt | I | Att |
| 449 | G | ggt | A | gCt |
| 458 | N | aat | Y | Tat |
| 474 | T | aca | I | aCa |
| 476 | M | atg | I | atA |
| 481 | A | gct | V | gTt |
| 493 | Y | tac | H | Tac |
| 508 | T | act | N | aCt |
| 527 | P | cct | T | Act |
| 533 | G | ggt | S | Agt |
| 537 | N | aat | I | aTt |
| 539 | R | aga | T | aCa |
| 543 | I | att | T | aCt |
| 553 | P | ccg | L | cTg |
| 561 | R | cgt | H | cAt |
| 568 | V | gtg | G | gGg |
| 574 | P | cct | L | cTt |
| 580 | C | tgt | Y | tAt |
| 584 | D | gat | V | gTt |
| 612 | E | gaa | D | gaT |
| 623 | S | agt | C | Tgt |
